# Serological evidence of acute rubella infection among under-fives in Mwanza: a threat to increasing rates of congenital rubella syndrome in Tanzania

**DOI:** 10.1186/s13052-016-0264-5

**Published:** 2016-05-25

**Authors:** Mariam M. Mirambo, Said Aboud, Martha F. Mushi, Mwanaisha Seugendo, Mtebe Majigo, Uwe Groß, Stephen E. Mshana

**Affiliations:** Department of Microbiology and Immunology, Weill Bugando School of Medicine, P.O. Box 1464, Mwanza, Tanzania; Department of Microbiology and Immunology, Muhimbili university of Health and allied sciences, P.O. Box 65001, Dar es Salaam, Tanzania; Department of Pediatrics and Child Health, Weill Bugando School of Medicine, P.O. Box 1464, Mwanza, Tanzania; Institute of Medical Microbiology, Gottingen University Medical Centre, Gottingen, Germany

**Keywords:** Rubella, IgM, Under-fives, Mwanza, Tanzania, Congenital rubella syndrome

## Abstract

**Background:**

Control of rubella infection is essential for preventing congenital rubella syndrome (CRS) and one of the important steps is to define a target population for vaccination. Therefore this study was done to determine serological evidence of acute rubella infection among under-fives in order to anticipate the magnitude of rubella virus transmission in Tanzania.

**Methods:**

A cross-sectional study involving children aged between 1 and 59 months was conducted between September and October 2014 before national rubella vaccination campaigns commenced. Rubella IgM antibodies were detected using commercial indirect enzyme-linked immunosorbent assay (ELISA). Data were analyzed using STATA version 11.

**Results:**

A total of230 under-fives were enrolled, their median age was 14 (Interquartile range (IQR) 7–26) months. The overall seroprevalence of rubella IgM antibodies was 10.9 % (25/230) with two confirmed cases of CRS. Two-sample Wilcoxon rank-sum test showed that the median age of rubella IgM seropositive children was significantly higher than that of IgM seronegative children (39 IQR: 18-51months vs. 14 IQR: 7–24 months, *P* < 0.001). On multivariate logistic regression analysis increase in age (OR: 1.07, 95 % CI; 1.03–1.1, *P* < 0.001) and residing in rural areas (OR: 8.07, 95 % CI; 1.43–45.6, *P* = 0.018) were independently found to predict acute rubella infection among under-fives.

**Conclusion:**

Our findings indicate that rubella virus is prevalent in our setting posing a risk of transmitting to childbearing aged women hence increasing the risk of CRS. Increasing prevalence of acute infection with age in under-fives indicates the protective role of maternal antibodies among infants. The sustained vaccination programme of under-fives as effective measure to control CRS should be emphasized in developing countries.

## Background

Rubella is a mild childhood illness transmitted through aerosol or direct contact. The disease affects mostly children, young adults, women of child bearing age and pregnant women [1]. It is common in many resource-constrained countries where vaccination has not yet been introduced. Rubella infection may present as an acute, mild or asymptomatic illness; therefore the outbreaks may occur without clinical recognition or may be misdiagnosed as measles cases [2, 3]. This poses difficulties for its clinical diagnosis, therefore serological tests for detection of specific rubella antibodies in suspected cases remains to be important [4]. In most cases, the disease is self-limiting and rarely causes complications in children. However, if contracted during the first trimester of pregnancy rubella virus may become a potential teratogenic agent which results into multiple organ defects referred to as the congenital rubella syndrome (CRS). More than 100,000 children are born with CRS each year especially in resource-constrained countries [3, 5, 6]. The majority of adolescents and women of child bearing age show high rubella seropositivity rates due to childhood exposure. Children usually harbour and spread the infection in a community including pregnant women which pose the risk to increasing rates of CRS [7]. Tanzania is among sub Saharan African country where rubella vaccine has been recently introduced and there are only few reports [8–11] on rubella; therefore the magnitude of rubella infection is unknown. This study was conducted to assess the magnitude of acute rubella infection among under-fives in order to understand the potential risk of transmitting to adults including pregnant women.

## Methods

A cross sectional hospital based study was carried out between end of September and October 2014 involving urban and rural settings of Mwanza city having a total population of about 7,064,453 [12]. The data were collected just before the national rubella vaccination campaign commenced in October 2014.

### Sample size and sampling procedures

The sample size was calculated using Kish Lisle formula of cross-sectional study. The prevalence of 31 % among under-fives from the study in Jos Nigeria was used [13]. The minimum sample size obtained was 226 under-fives. Three reproductive and child health clinics (RCH) representing urban and rural settings of Mwanza city were conveniently selected. Under-fives were serially and proportionally enrolled from Makongoro and Bugando (urban); and Karume (rural) until the desired sample size was reached. 

### Data collection and laboratory procedures

After obtaining written informed consent from parent/guardian, 2 – 4 ml of blood samples were collected using plain vacutainer tubes (Becton Dickinson, Nairobi, Kenya) and transported to the Bugando multipurpose laboratory whereby sera were separated and stored in cryovials at −80 °C freezer until the time for processing. Standardized data collection tool was used to obtain social demographic information and the presence or absence of rashes was noted. Data collected included age, sex, residence, socio-economic status, number of siblings, and presence of rashes or family member with rash. Good social economic status was defined as parents being educated (secondary education and above) with sustainable income (>1USD/day) or income generating activities such as tailoring, fishing, petty business etc.

### Laboratory investigations

Sera were tested for presence of specific IgM usingcommercial indirect enzyme-linked immunosorbent assay (ELISA) (ChemWell® 2910-Awareness Technology Inc., Palm City, USA) according to the manufacturer’s instructions with the positive cut off values of ≥ 1 index. ELISA has been found to be a reliable method for assaying rubella antibodies with sensitivity and specificity of >97 % [14].

### Data management and analysis

Data were entered into a computer using Microsoft Office Excel 2007 and analyzed using the STATA version 11 (College Station, Texas, USA). Categorical variables were presented as proportions and analyzed using the Pearson’s Chi-square to observe the differences among the various groups. Continuous variables were summarized as median with interquartile ranges. Univariate analysis and multivariate logistic regression models were fitted to determine factors associated with rubella IgM seropositivity. We used a backward-stepwise selection model to select factors with a p-value <0.5 to be fitted into the multivariate logistic regression analysis. Odds ratio (OR) and 95 % confidence interval [95 % CI] were presented.

### Ethical clearance

The protocol for conducting the study was approved by the Joint Catholic University of Health and Allied Sciences/Bugando Medical Centre (CUHAS/BMC) research ethics and review committee. Permission was sought from hospital/clinics administration. Written informed consent was obtained from each parent/guardian prior recruitment to the study. All under-fives with acute infection were further assessed bypediatrician to ascertain features of CRS.

## Results

### Social demographic characteristics

A total number of 230 under-fives were enrolled with the median age of 14 (Interquartile range (IQR) 7–26) months. Age distribution of under-fives were 90 (39 %), 68 (29 %), 32 (13.9 %), 13 (5.6 %) and 27 (11.7 %) for 0–11, 12–23, 24–35, 36–47 and 48–59 months respectively. Of the 230 under-fives, 65 (28.3 %), 118 (51.3 %) and 47 (20.4 %) were enrolled from Bugando Medical Centre- pediatric outpatients Department, Karume and Makongoro clinics, respectively. A total of 86 (37.4 %) and 144 (62.6 %) were residing in urban and rural areas, respectively. A total number of 166 (72.2 %) and 64 (27.8 %) were from families with good and poor socio-economic status, respectively. Of the under-fives, 116 (50.4 %) and 114 (49.6 %) were females and males respectively. There was no significant difference in the median number of siblings between the children residing in rural and those residing in urban areas (2; IQR:2–3 vs. 2;IQR:2–4, *P* > 0.05).

### Prevalence of rubella-specific IgM antibodies and associated factors

**Fig. 1:**
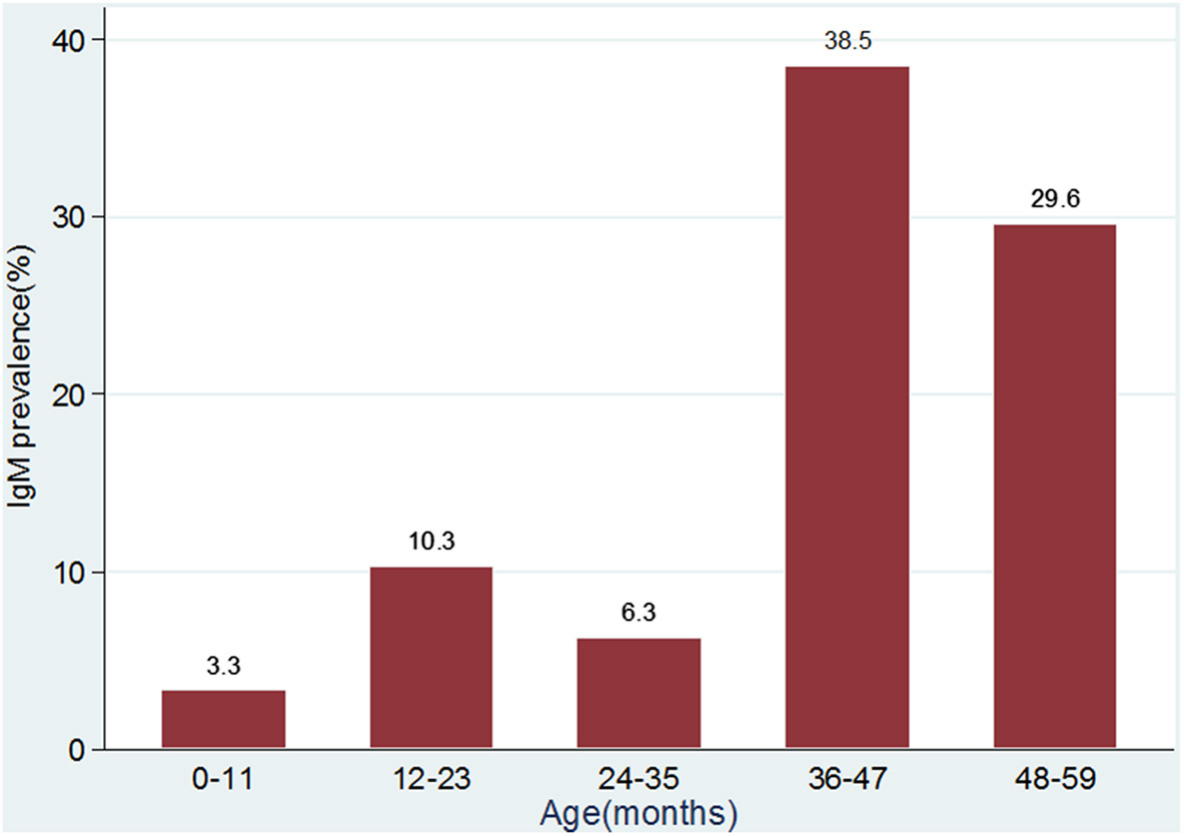
Age specific prevalence of rubella IgM antibodies among under-5 children in Mwanza city

Overall, seroprevalence of rubella IgM antibodies was 10.9 % (25/230) with median index value of 1.4: IQR: 1.2–2.5. The median age for IgM seropositive children was 39 (IQR: 18–51) months while that of IgM seronegative was 14 (IQR:7–24) months and the difference was statistically significant (*P* < 0.001). The rubella IgM seroprevalence was 3 (3.3 %), 7 (10.3 %), 2 (6.3 %), 5 (38.5 %) and 8 (29.6 %) among children aged 0–11, 12–23, 24–35, 36–47 and 48–59 months, respectively (Fig. 1). The seroprevalence of rubella IgM was found to increase significantly as the age increases (OR: 1.05 95 % CI: 1.03–1.08, *P* < 0.001). On univariate analysis; as the number of siblings increases the seroprevalence of rubella IgM tends to decrease significantly (OR: 0.6 95 % CI: 0.36–0.99, *P* = 0.044). The risk of having acute rubella infection was higher among under-fives presented with rash. However the level of significance was borderline (OR:2.18, 95 % CI: 0.92–5.17, *P* = 0.07).On multivariate logistic regression analysis, increase in age by a month and residing in rural areas were found to be independent predictors of having acute rubella infection (Table 1).

**Table 1:** Univariate and multivariate regression analyses of factors associated with rubella IgM seropositivity among children in Mwanza city

Characteristics	IgM seropositivity (N, %)	Univariate		Multivariate	
		OR (95 % CI)	*P* value	OR (95 % CI)	*P* value
Age (months)	39 (IQR:18–51)	1.05 (1.03–1.08)	<0.001	1.07 (1.03–1.10)	<0.001
Number of Siblings	2 (IQR:2–2)	0.6 (0.36–0.99)	0.044	0.57 (0.31–1.04)	0.069
Sex					
Female (116)	13 (11.4 %)	1			
Male (114)	12 (14.2 %)	0.89 (0.39–2.05)	0.790	1.15 (0.35–3.79)	0.807
Residence					
Urban (86)	7 (8.14 %)	1			
Rural (144)	18 (12.5 %)	1.612 (0.64–4.03)	0.307	8.07 (1.43–45.64)	0.018
Socio-economic status					
Good (166)	21 (12.7 %)	1			
Poor (64)	4 (6.3 %)	0.46 (0.17–1.39)	0.171	0.53 (0.11–2.34)	0.400
Rash					
No (172)	15 (8.7 %)	1			
Yes (58)	10 (17.2 %)	2.18 (0.92–5.17)	0.077	0.903 (0.24–3.34)	0.879
Blood transfusion					
No (216)	23 (10.7 %)	1			
Yes (14)	2 (14.3 %)	1.398 (0.29–6.64)	0.673		

### Clinical assessment of IgM positive children

Out of 25 IgM positive under-fives 3 were infants. Of 3 infants, 2 (one at 4 months and another at 6 months) were confirmed to have features of CRS both had congenital heart defects and congenital cataracts.

## Discussion

Control of rubella infection is crucial for eliminating CRS. However, the control should be strategic and cost effective to achieve a milestone of preventing CRS. One of the strategies to accomplish this is to define the target population for vaccination and maintain surveillance of CRS. There are few reports on acute rubella infection among under-fives in Africa [7]. This study for the first time in Tanzania provides information regarding acute rubella infection among under-fives; the group that can be source of infection to pregnant women.

The seroprevalence of acute rubella infection among the under-fives was found to be comparable with previous study in India [15] but significantly higher compared to another study which was done a year later in India among pediatric population before vaccination era [16]. However, the magnitude of acute infection among under-fives in the current study is lower than that reported from Nigeria among under-fives [13]. Given the fact that in developed countries a single rubella case is considered as a potential risk for transmitting rubella virus to pregnant women which may results into poor pregnancy outcome; the reported prevalence in this study is indeed high [17]. This is further supported by our findings whereby 2 out 230 under-fives in the current study were confirmed to have CRS.

Previous studies in the same setting observed high prevalence of natural immunity against rubella virus among pregnant women [11, 18]. Maternal rubella antibodies have been found to provide protection against acute rubella infection in the first six months of life. The role of maternal antibodies in the protection of acute rubella infection was confirmed in the current study as evidenced by the fact that as the age increases the risk of having acute rubella infection was found to increase significantly [19].

Children residing in rural areas were found to be at high risk of having acute rubella infection as compared to the urban group. Most of houses in the rural areas of Mwanza are not well ventilated hence pose a potential risk for transmitting airborne diseases as previously documented [20]. In the current study though not significant on multivariate analysis; as the number of siblings increases the risk of having acute rubella infection decreases. Due to the nature of the disease high transmission rates occurs in congested areas. Therefore; children from households with high number of siblings might have previously exposed to rubella viruses therefore they are naturally protected [17, 21–23].

## Conclusion

In conclusion acute rubella infection among under-fives in Mwanza is high with about one out of 100 under-fives having CRS. Among under-fives increase in age and residing in rural areas were independent factors predicting acute rubella infection. Acute rubella infection among under-fives poses a potential risks to susceptible pregnant women which often results into poor pregnancy outcome including CRS. The findings support the current programme of vaccinating all under-fives. In addition; screening and vaccinating susceptible childbearing aged women should be considered in developing countries in efforts to control CRS.
